# Influence of the Different Maturation Conditions of Cocoa Beans on the Chemical Profile of Craft Chocolates

**DOI:** 10.3390/foods13071031

**Published:** 2024-03-28

**Authors:** Ana Carolina do Carmo Mazzinghy, Viviane Dias Medeiros Silva, Ana Luiza Coeli Cruz Ramos, Carla Patrícia de Oliveira, Gabriel Barbosa de Oliveira, Rodinei Augusti, Raquel Linhares Bello de Araújo, Júlio Onésio Ferreira Melo

**Affiliations:** 1 Campus Sete Lagoas, Universidade Federal de São João del-Rei, Sete Lagoas 35701-970, MG, Brazil; anamazzinghy@yahoo.com.br (A.C.d.C.M.); vivianedms05@gmail.com (V.D.M.S.); carlapatoli@ufsj.edu.br (C.P.d.O.); 2 Departamento de Alimentos, Universidade Federal de Minas Gerais, Belo Horizonte 31270-901, MG, Brazil; analuizacoeli@gmail.com (A.L.C.C.R.); biohits@gmail.com (G.B.d.O.); raquel@bromatologiaufmg.com.br (R.L.B.d.A.); 3 Departamento de Química, Universidade Federal de Minas Gerais, Belo Horizonte 31270-901, MG, Brazil; augusti.rodinei@gmail.com

**Keywords:** *Theobroma cacao*, maturation, chemical profile

## Abstract

Cocoa beans (*Theobroma cacao* L.) can be used for craft chocolate production, which arouses consumer interest due to their perceived better quality. This study aimed to evaluate the chemical profile of 80% artisanal chocolate samples produced with cocoa beans subjected to different maturation conditions. In the first maturation process, beans were matured under no-oxygen conditions, and in the second, the toasted beans were matured in oak barrels. The volatile compounds of the chocolate samples were extracted by the solid-phase microextraction method in headspace mode and analyzed by gas chromatography/mass spectrometer. The non-volatile compounds were extracted with methanol and analyzed through paper spray mass spectrometry. Overall, 35 volatile compounds belonging to different chemical classes (acids, alcohols, aldehydes, ketones, esters, and pyrazines) were identified, such as propanoic acid and butane-2,3-diol. In addition, 37 non-volatile compounds, such as procyanidin A pentoside and soyasaponin B, were listed. Tannins, flavonoids, and phenylpropanoids were the main chemical classes observed, varying between the two samples analyzed. Therefore, it was possible to verify that maturation conditions affected the metabolomic profile of the 80% artisanal chocolate samples, being able to influence the sensory characteristics and bioactive compounds profile. Given these results, the sensory evaluation of these chocolates is suggested as the next step.

## 1. Introduction

Cocoa (*Theobroma cacao* L.) is the fruit of the cocoa tree, which is a small tree [1,2] from the Malvaceae family and is native to tropical America [3,4]. This species has different varieties, being called Forastero, Criollo, and Trinitario, and can be identified by checking the size, shape, and attributes, such as the color and appearance of the fruit, in addition to the geographical origin of the fruit [5,6]. Cocoa comprises the peel, pulp, and seed and can contain up to 40 seeds [7,8]. Cocoa is a versatile fruit with significant economic importance due to its vast industrial applicability [9]. However, its primary use is directed to manufacture chocolates [10,11]. To produce chocolate, cocoa beans are used exclusively [12]. These are considerably important for the food industry, as they are the parts that most add economic value to the fruit [13,14]. Cocoa products with unique characteristics are the most sought after in the market due to their uniqueness, related to the composition and the specific flavor [11].

Recently, the production of craft chocolates has been arousing interest worldwide. The production method of these chocolates manages the entire production process, which goes from obtaining the cocoa bean to manufacturing the chocolate bars [15,16]. These chocolates are produced by artisanal methods using high-quality cocoa beans, which, after being harvested, are subjected to fermentation, drying, and roasting [17,18]. Fermentation is crucial for developing flavor precursors and bioactive compounds characteristic of cocoa and its derivatives [7,11]. Although it is not mandatory in processing, one step that is being implemented by the companies that produce these artisanal chocolates is the maturation of the beans. According to Castro et al. [19], barrel maturation aims to confer a sensory complexity to food. During this stage, the extraction of phenolic compounds occurs, in addition to a series of chemical reactions such as hydrolysis, oxidation, polymerization, and esterification.

Chocolate has a unique flavor and aroma, and these sensory attributes are related to the diversity of volatile and non-volatile compounds present in the composition of cocoa [20,21]. The volatile compounds confer various aromas to the products, being perceived by the retro-nasal and gustatory senses [22]. These aroma characteristics, along with the color and texture of the chocolate, are fundamental in the perception and indicate consumer preference [23,24,25].

The consumption of cocoa and chocolate is related to several beneficial effects on human health due to the presence of substances that give them this potential. The main benefits reported are related to the prevention of cardiovascular diseases, improvement of glucose homeostasis, prevention of obesity, and improvement of the immune system, in addition to evidence regarding the central nervous system [26,27].

The cocoa is processed in several stages, from the fruit harvest to obtaining cocoa butter; subsequently, the chocolate is produced [28,29]. Therefore, it is important to investigate the influence of maturation conditions on the chemical characteristics of this raw material and its products. Several modern methods can characterize complex matrices, such as food. One of these methods is headspace solid-phase microextraction (HS-SPME), which has been constantly used for food analysis to determine volatile compounds. HS-SPME avoids the need to use organic solvents and the destruction of sample analytes [23,30].

Some compounds are not volatile. However, they can be analyzed using paper spray mass spectrometry (PS-MS), which is a technique that has been widely used for the rapid acquisition of the fingerprints of complex substances, such as drugs and metabolites, due to being versatile, simple, and low-cost, in addition to demonstrating greater applicability in food analysis [31,32].

This study aimed to evaluate the chemical profile in the non-volatile and volatile composition of 80% artisanal chocolate samples, in which the cocoa beans used were submitted to two different maturation conditions.

## 2. Materials and Methods

### 2.1. Samples

The artisanal chocolate samples used in this study were provided by the company Miller and Fields Craft Chocolate, located in Florianópolis, Brazil, and the cocoa used to manufacture these chocolates was originally from Pará, Brazil. The two chocolate samples contained 80% cocoa, with a difference between the cocoa bean maturation conditions. Sample 1 (beans): Matured in a 200 L food container with a sealing lid and in the absence of oxygen. After a few months, the natural formation of a vacuum was observed due to the natural respiration of the cocoa. Sample 2 (toasted beans): Matured in a French oak barrel. Both samples were matured at room temperature and in relative humidity between 70 and 80%. After two years of maturation, the samples were roasted and ground (stone mill) under the same conditions. To manufacture these chocolates, a blend of the cocoa varieties Forastero, Trinitario (a hybrid between Criollo and Forastero), and some cocoa clones were used.

### 2.2. Extraction and Identification of Volatile Organic Compound

The solid-phase microextraction method in headspace mode (HS-SPME) was used for the extraction of the volatile organic compounds (VOCs) with polydimethylsiloxane-divinylbenzene (PDMS/DVB) (65 μm) fiber, as described by Ramos et al. [30].

Initially, 2.0 g of chocolate samples were weighed in a 20 mL headspace vial and sealed with an aluminum seal in triplicate. Subsequently, the samples were preheated on a heating plate for 5 min. After preheating, the PDMS/DVB fiber was inserted into the vial and exposed to the chocolate samples for 10 min. This fiber was removed from the vial and manually inserted into the GC-MS, in which the temperature in the injector was 250 °C, with a desorption time of 5 min, the temperature of the ion source was 200 °C, and the interface was 275 °C.

VOC identification was performed on a gas chromatograph (Agilent 7890 B) coupled with a mass spectrometer (Agilent 5977A) with a quadrupole analyzer with a split/splitless injector in splitless mode. For this, the following conditions were applied: injector temperature of 250 °C, ion source temperature of 275 °C, and interface temperature of 275 °C. Ultrapure helium gas was used as a carrier gas at a flow rate of 1 mL min^−1^, and the VOCs were separated using a capillary column Varian Cp-Wax 52CB (CP 8714), 30 m × 0.25 mm × 0.25 μm (Agilent Technologies).

The initial heating of the column was 40 °C for 1 min, increasing from 20 °C min^−1^ up to 70 °C and remaining at the temperature for 1 min, then 8 °C min^−1^ up to 100 °C remaining for 3 min. There was an increase from 10 °C min^−1^ to 150 °C remaining for 1 min. Finally, the temperature increased to 250 °C, remaining for 3 min. The total running time was 27 min. Data acquisition was generated in full-scan mode with a range of 35 to 350 *m*/*z*, obtained by the electron impact ionization (EI) technique at an energy of 70 eV.

The spectra of the VOCs were identified according to their fragmentation profile, which was compared with the mass spectra of the National Institute of Standards and Technology (NIST) library, using a similarity level greater than 80%. In addition, the data were confirmed by comparing them with the compounds already reported in the literature [23,30,33,34].

### 2.3. Extraction and Paper Spray Mass Spectrometry Analysis

Previously crushed and homogenized chocolate samples were weighed (1.0 g) and mixed with 8 mL of HPLC-grade methanol in triplicate. Then, they were shaken for 30 s and kept at rest for one hour at ambient temperature (25 °C). The chocolate extracts’ analysis with PS-MS was carried out on an LCQ mass spectrometer (Thermo Scientific, San Jose, CA, USA) equipped with a paper spray ionization source in two ionization modes: positive and negative.

The instrumental conditions of the PS-MS analysis were: voltage applied to the paper, 4.5 kV (positive mode) and 3.5 kV (negative mode); capillary temperature, 275 °C; capillary voltage, 40 V; tube lens voltage, 120 V. Full-scan mass spectra were acquired in a range of 100 to 1000 *m/z* (charge mass ratio). The chromatographic paper was cut in a triangular shape with a dimension of 1.0 × 1.5 × 1.5 cm [35,36].

The extracts of the chocolate samples (2 μL) were applied to the triangular base of the paper, followed by the addition of 40 μL of methanol, and the voltage was established through the metal clip. Subsequent fragmentations were performed with the collision energy between 15 and 45 eV. The mass spectra data were processed using Xcalibur software version 2.1 (Thermo Scientific, San Jose, CA, USA). For future analysis, the average mass spectra were listed and organized through spreadsheet software (Excel, 2020, Microsoft, Redmond, WA, USA). The metabolites’ identification, their *m/z*, and fragments were compared with the data previously found in the literature [35,36,37].

## 3. Results

### 3.1. Volatile Organic Compound Profile

In the 80% chocolate samples, 35 volatile compounds were found using the PDMS/DVB fiber (Table 1), of which 26 were identified in Sample 1 and 20 in Sample 2.

### 3.2. Paper Spray Mass Spectrometry Analysis

**Figure 1:**
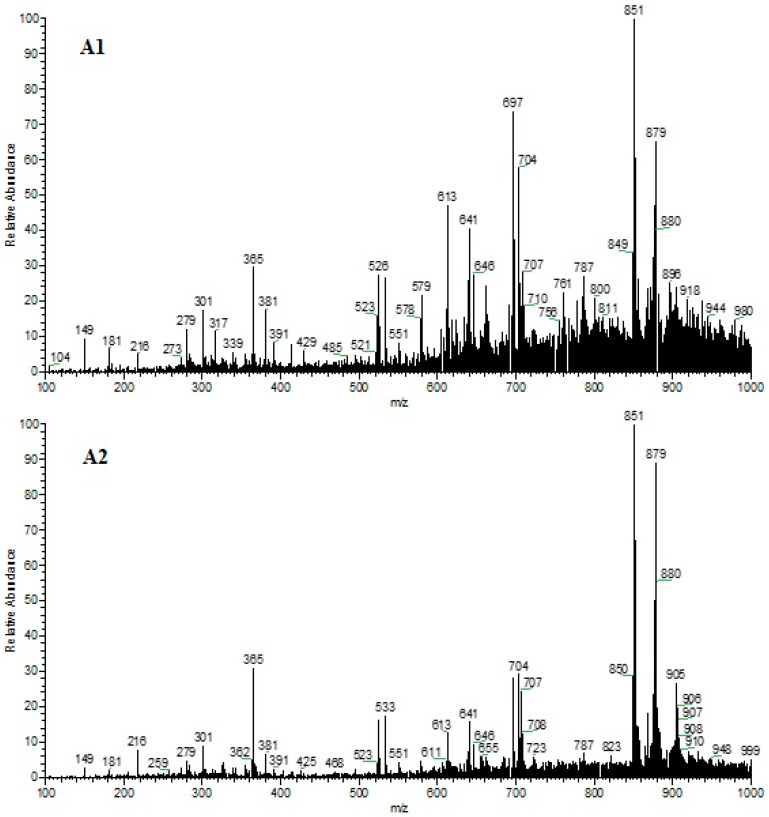
Representation of the spectra of chocolate Sample 1 (**A1**) and Sample 2 (**A2**), for the positive ionization mode.

**Figure 2:**
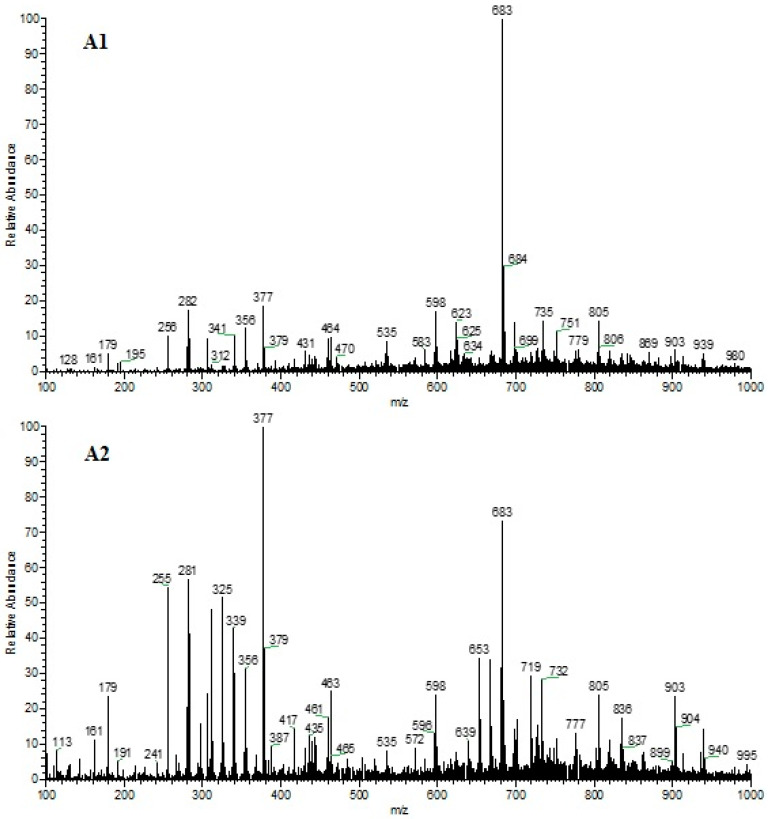
Representation of the spectra of chocolate Sample 1 (**A1**) and Sample 2 (**A2**) for the negative ionization mode.

The spectra referring to the chemical profile of the fixed compounds of the two 80% chocolate samples in the positive and negative ionization modes can be observed in Figure 1 and Figure 2, respectively.

Through the identification attempt, it was possible to find 37 compounds analyzing their Figures S1–S4 spectra, of which 33 were identified in the negative ionization mode and four in the positive ionization mode (Table 2).

## 4. Discussion

### 4.1. Volatile Organic Compound Profile

In the present study, the chemical classes found in the chocolate samples were acids, alcohols, aldehydes, ketones, phenylpropanoids, esters, and pyrazines. When conducting a study on the volatile composition of seven chocolate samples, Waehrens et al. [49] identified approximately 69 compounds, which also belonged predominantly to these chemical classes. In addition, the authors emphasized that most of these compounds were identified in chocolates whose cocoa composition was higher than 70%. These results support the compounds’ presence in these samples, considering that the chocolates evaluated had 80% cocoa in their composition.

Hexadecanoic acid (Table 1) was also identified by Kouassi et al. [50] when evaluating the volatile composition of cocoa and the sensory perceptions of chocolate. As Ascrizzi et al. [51] reported, the acids (fatty, organic, and others) are evaporated in the drying stages of cocoa beans due to their volatility because the peel surrounding the beans is permeable.

However, it is essential to note that these acids confer a mild and pleasant flavor to chocolate. On the other hand, Chagas Junior et al. [52] warn that the presence of large quantities of acids in cocoa beans may confer undesirable characteristics to the quality of the chocolate, such as susceptibility to oxidative rancidity, which affects the product’s shelf life.

In both Sample 1 and Sample 2, the compound butane-2,3-diol was identified. Tuenter et al. [39], when evaluating samples of cocoa liquor and fine chocolate using the same method used here, found that alcohols corresponded to the second predominant chemical class in both samples evaluated, which corroborates the present results. In addition, these researchers also identified the presence of the compound 2,3-butanediol. Rodriguez-Campos et al. [53] also detected the same compound in cocoa beans during the fermentation and drying process. In their analysis of chocolates made with cocoa from different countries, Calva-Estrada et al. [54] reported that alcohols are of significant importance in the volatile chemical profile of chocolate since they have the potential to confer aroma to chocolate. All of the authors describe these compounds as being desirable to obtain a quality final product with specific sensory characteristics of chocolate.

Only in Sample 2, where the toasted beans matured in a French oak barrel, were the following aldehydes identified: benzaldehyde, 5-methyl-2-phenyl-2-hexenal, and 3-methyl hexanal. Benzene acetaldehyde and nonanal were identified in both samples. According to Kouassi et al. [50] and Hamdouche et al. [55], aldehydes result from oxidative reactions and fatty acid degradation during the fermentation process of cocoa beans. The beans’ maturation under the absence of oxygen may have contributed to the non-production of these four aldehydes identified only in Sample 2. Aldehydes are desirable since they are responsible for giving fruity and floral notes to cocoa products, as explained by Cemin et al. [20].

In this study, two ketones were detected: ethanone and acetoin. Kouassi et al. [50] warn that high ketone contents may be conducive to obtaining an excellent aromatic quality of cocoa after the fermentation stage. Esters are compounds that give a fruity aroma to cocoa beans [56]. In this study, it was possible to verify the presence of acetic acid-2-phenylethyl-ester in the two studied samples, but hexadecanoic acid methyl ester was only present in Sample 2. According to Kouassi et al. [50], these compounds are higher in fresh cocoa beans. This result suggests that maturation in oak barrels maintains the beans’ freshness since hexadecanoic acid methyl ester was identified in the final product.

According to Chagas Junior et al. [52], the fermentation stage of cocoa beans, as well as the later stages, are crucial for the sensory characteristics of the final product, which may influence the volatile composition of the final product, as reported by Afoakwa et al. [15]. Therefore, any alteration in the cocoa beans’ processing conditions may interfere with the chemical composition of the chocolate. With this information, these variations related to the identified chemical classes were expected, taking into account the different maturation conditions of the beans.

Even though these differences occurred, it is noticeable that the chemical classes found corroborate other findings in the literature, which also identified these classes in studies involving the chemical profile of cocoa and/or chocolate products [20,38,39,40].

### 4.2. Paper Spray Mass Spectrometry Analysis

In Sample 2, in which the toasted beans were matured in a French oak barrel, it was possible to detect the presence of hydroxy octadecenedioic fatty acid. This result was similar to that found by Greño et al. [41], Ramos-Escudero et al. [57], and Oliveira Júnior et al. [4], who also identified these compounds in cocoa beans and chocolates. However, Melo et al. [58] observed that the fatty acid profile varies according to the type of chocolate and the percentage of cocoa. According to Nagy and Tiuca [59], fatty acids, in general, are components of great relevance for human health because they have various biological and structural activities. Thus, the presence of these in the chemical composition of chocolate becomes desirable.

A disaccharide (glucose) was identified only in Sample 2, while hexitol was identified in Sample 1, where the cocoa beans were matured under oxygen-free conditions. Lavorgna et al. [42], when evaluating the chemical profile of cocoa beans of the Criollo variety, also detected disaccharide and hexitol compounds. These sugars are commonly found in chocolates, as they are added ingredients to obtain the sensorily desirable final product [60].

Serotonin was identified in the two chocolate samples evaluated. Wang et al. [61], when evaluating cocoa seeds, found this same compound. According to these authors, cocoa, as well as its products, have been arousing interest due to the potential beneficial effect on human health, among them, antidepressant effects due to high levels of serotonin. Therefore, this suggests that these chocolate samples have the potential effect of combating depression, regardless of the maturation conditions of the cocoa beans. According to Sezini and Coutto Gil [62], the benefits of serotonin are not limited to the central nervous system, given that this compound acts on other tissues, such as the gastrointestinal and cardiovascular.

The amino acid tryptophan was identified only in Sample 2, corroborating the works of Oliveira Júnior et al. [4] and Rosa et al. [45], who also identified this compound in cocoa beans and their products. According to Oliveira Júnior et al. [4], this amino acid is essential because it is the precursor of serotonin. However, its concentration in plasma is regulated through the balance of its intake in the diet and its removal from plasma for protein synthesis. Barros et al. [63] point out that chocolate with 70% cocoa, when consumed in the diet correctly, is fundamental to improving the central nervous system and promoting feelings of pleasure and well-being.

It was possible to detect some flavonoids derived from quercetin, procyanidins, and apigenin. Seem et al. [64] highlight that the flavonoids present in cocoa have potential benefits for bone health and exert antioxidant and anti-inflammatory activities. Murphy et al. [65] reported that flavonoids and their subclasses are found in several fruits. However, they are predominantly found in cocoa and its products. Thus, it is possible to infer that most of the flavonoids were identified in the chocolates in which the toasted beans were maturated in a French oak barrel, evidencing that this stage of maturation, as well as the analogous conditions of this container, was probably favorable for the synthesis of these compounds. Quiroz-Reyes and Aguilar-Méndez [66] also point out that it is common to find this chemical class in cocoa derivatives. Given the above, the similarity with the results obtained from this study is noticeable.

## 5. Conclusions

Solid-phase microextraction coupled with mass spectrometry was efficient, separating and identifying 35 compounds belonging to several chemical classes. It was possible to verify that in Sample 2, in which the toasted beans matured in a French oak barrel, there was a predominance of aldehydes, which can be considered desirable for chocolates since these compounds are responsible for conferring fruity and floral notes to the products derived from cocoa, evidencing the modification of the chemical profile of chocolate, according to the conditions analogous to the processing step.

The analysis of the chemical profile using PS-MS was efficient since 37 compounds were suggested. The main class found in both samples was flavonoids, but mostly in Sample 2. While the phenylpropanoids were identified only in Sample 2, evidencing that the toasting of beans and the maturation in an oak barrel altered the sensory profile of the chocolate as well as the profile of bioactive compounds. These results can be considered important and desirable for the food industry, considering that these new steps implemented during the processing of chocolates can be viable alternatives for the search for new flavors and aromas associated with the quality of these foods.

## Figures and Tables

**Table 1:** Volatile compounds found in 80% chocolate samples, using PDMS/DVB fiber through the SPME/CG-MS technique.

N°	Compound	Formula	CAS	Sample 1	(%) Area	Sample 2	(%) Area	Reference
**Fatty acid**								
1	9-octadecenoic acid	C_18_H_34_O_2_	112-79-8	X	1.72	X	5.70	[38]
2	Decanoic acid	C_10_H_20_O_2_	334-48-5	ND		X	3.51	[20]
3	3-hydroxydecanoic acid	C_12_H_24_O_3_	1883-13-2	ND		X	0.73	[20]
4	Dodecanoic acid	C_12_H_24_O_2_	143-07-7	ND		X	0.65	[20]
5	Cyclopropanetetradecanoic acid	C_26_H_50_O_2_	27198-62-5	X	1.19	ND		[20]
6	Eicosanoic acid	C_20_H_40_O_2_	506-30-9	X	0.79	ND		[20]
7	Oleic acid	C_18_H_34_O_2_	112-80-1	X	4.46	ND		[20]
8	Hexadecanoic acid ethyl ester	C_18_H_36_NO_2_	658-97-7	ND		X	10.61	[20]
**Organic acid**								
9	Acetic acid	C_2_H_4_O_2_	64-19-7	X	25.68	X	39.23	-
10	Benzene acetic acid	C_8_H_8_O_2_	103-82-2	X	12.33	X	3.58	-
11	Benzoic acid	C_7_H_6_O_2_	65-85-0	X	4.35	X	1.50	-
12	3-methyl-butanoic acid	C_5_H_10_O_2_	503-74-2	X	3.38	ND		-
13	Nonanoic acid	C_9_H_18_O_2_	112-05-0	X	2.24	X	4.43	-
14	Phthalic acid	C_8_H_6_O_4_	88-99-3	X	3.03	ND		-
15	Propanoic acid	C_3_H_6_O_2_	79-09-4	X	0.77	X	0.68	-
**Alcohol**								
16	1-hexanol-2-ethyl	C_8_H_18_O	104-76-7	X	0.86	ND		[20]
17	Butane-2,3-diol	C_4_H_10_O_2_	513-85-9	X	7.94	X	4.95	[20]
18	2-dodecanol	C_12_H_26_O	10203-28-8	X	1.95	ND		[20]
19	Phenylethyl alcohol	C_8_H_10_O	60-12-8	X	1.84	X	1.00	[20]
**Aldehyde**								
20	5-methyl-2-phenyl-2-hexenal	C_13_H_16_O	21834-92-4	ND		X	0.61	[38]
21	Benzene acetaldehyde	C_8_H_8_O	122-78-1	X	1.19	ND		[38]
22	Benzaldehyde	C_7_H_6_O	100-52-7	ND		X	1.52	[38]
23	Nonanal	C_9_H_18_O	124-19-6	X	13.16	X	13.89	[38]
24	3-methyl hexanal	C_7_H_14_O	19269-28-4	ND		X	0.94	[38]
**Ketone**								
25	Acetoin	C_4_H_8_O_2_	513-86-0	X	1.35	ND		[20]
26	Ethanone	C_2_H_2_O		X	1.66	X	0.62	[20]
**Benzoic acid derivatives**								
27	1-2-4-enzenetricarboxylic acid	C_9_H_6_O_6_	528-44-9	X	1.27	ND		[38]
28	2-5-dihydroxybenzaldehyde	C_7_H_6_O_3_	1194-98-5	X	0.65	ND		[38]
**Ester**								
29	Acetic acid-2-phenylethyl-ester	C_10_H_12_O_2_	103-45-7	X	2.68	X	1.30	[39]
30	Hexadecanoic acid methyl ester	C_17_H_34_O_2_	112-39-0	ND		X	0.98	[39]
**Phenylpropanoid**								
31	N-benzyl-2-aminociannamate	C_17_H_17_NO_2_	18429-69-1	X	2.20	ND		-
**Pyrazine**								
32	Pyrazine tetramethyl	C_8_H_12_N_2_	1124-11-4	X	0.91	ND		[40]
33	Pyrazine	C_4_H_4_N_2_	290-37-9	ND		X	3.58	[40]
**Others**								
34	4H-pyran-4-2-3 dihydro 3,5-dihydroxy-6-methyl	C_5_H_6_O_2_	28564-83-2	X	1.93	ND		-
35	3H-pyrazol-3-one, 2,4-dihydro-5-methyl-2-phenyl-	C_10_H_10_N_2_O	89-25-8	X	0.48	ND		-

X = Compound identified; ND = Not identified.

**Table 2:** Proposed assignments for ions detected in chocolate samples by PS-MS.

N°	Identification Attempt	*m*/*z*	MS/MS	ID	Sample 1	Sample 2	Reference
**Fatty acids**							
1	Hydroxy octadecenedioic	327	171, 211, 229	[M–H]^−^	ND	X	[41]
Sugar							
2	Disaccharide	341	341, 236, 198	[M–H]^−^	ND	X	[42]
3	Glucose	179	161, 113, 89	[M–H]^−^	ND	X	[43]
4	Hexitol	181	181, 113, 101	[M–H]^−^	X	ND	[42]
5	*β*-D-xylopyranosyl-α-L-rhamnopyranosyl-D-fucose	441	-	[M–H]^−^	ND	X	[42]
**Amino acids and derivaties**							
6	l-tryptophan	205	146, 188, 184	[M+H]^+^	ND	X	[44]
7	Serotonin	149	132, 136, 118	[M+H]^+^	X	X	[44]
**Benzoic acid derivatives**							
8	Vanillic acid diglucoside	461	353, 353, 123	[M–H]^−^	ND	X	[4]
9	20-hydroxyecdysone-3-O-*β*-d-xylose	579	-	[M+H]^+^	X	X	[45]
**Phenylpropanoid**							
10	Caffeoyl tyrosine	354	342, 298, 256	[M–H]^−^	ND	X	[43]
11	Dideoxyclovamide	342	147, 119, 120	[M–H]^−^	ND	X	[43]
12	Epigallocatechin	305	289, 151, 169	[M–H]^−^	ND	X	[46,47]
13	12-hydroxy-jasmonic acid	225	-	[M–H]^−^	ND	X	[41]
**Flavonoids**							
14	Apigenin-7-O-glucoside	578	577, 269	[M–H]^−^	X	ND	[48]
15	Apigenin-8-C-glucoside	432	431, 341, 311	[M–H]^−^	ND	X	[48]
16	Dimethyl-O-EC-EC-ECG trimer	909	-	[M–H]^−^	ND	X	[48]
17	Dimethyl-O-EC-ECG dimer	621	-	[M–H]^−^	ND	X	[48]
18	Naringenin-7-O-neohesperidoside	580	579, 459, 271	[M–H]^−^	X	ND	[48]
19	Quercetin-3-O-arabinoside	433	383, 301, 139	[M–H]^−^	ND	X	[4]
20	Quercetin-3-O-galactoside	464	463, 301	[M–H]^−^	ND	X	[48]
21	Quercetina-3-O-*β*-d-glucopyranosside	463	107, 121, 151	[M–H]^−^	ND	X	[48]
**Tannins and precursors**							
22	Procyanidin A-type pentamer arabinoside	785	591, 547, 439	[M–H]^−^	X	ND	[43]
23	Procyanidin 2A-type trimer	861	575, 425, 289	[M–H]^−^	X	X	[48]
24	Procyanidin A hexoside;	737	611, 585, 539	[M–H]^−^	ND	X	[48]
25	Procyanidin A pentoside	707	581, 539, 449	[M–H]^−^	X	X	[48]
26	Procyanidin A-type pentamer	719	-	[M–H]^−^	ND	X	[4]
27	Procyanidin A-type tetramer arabinoside	641	-	[M–H]^−^	ND	X	[48]
28	Procyanidin A-type tetramer hexoside	656	-	[M–H]^−^	ND	X	[48]
29	Procyanidin A-type trimer	865	739, 713, 695	[M–H]^−^	ND	X	[48]
30	Procyanidin B dimer	577	425, 407, 289	[M–H]^−^	ND	X	[48]
31	Procyanidin A-type hexamer	864	-	[M–H]^+^	X	ND	[43]
32	Procyanidin A-type trimer	863	-	[M–H]^−^	X	ND	[43]
33	Dimethyl-O-procyanidin B trime	893	-	[M–H]^−^	X	ND	[4]
34	Procyanidin A-type hexamer arabinoside	929	739, 713, 695	[M–H]^−^	X	ND	[4]
35	Procyanidin trimer	944	695, 577, 425	[M–H]^−^	X	ND	[4]
**Terpenoids**							
36	Soyasaponin B I	941	615, 733, 879	[M–H]^−^	X	X	[41]
37	Soyasaponin B II	911	695, 577, 425	[M–H]^−^	X	ND	[41]

X = Compound identified; ND = Not identified.

## Data Availability

The original contributions presented in the study are included in the article/Supplementary Material, further inquiries can be directed to the corresponding author.

## Supplementary Materials

The following supporting information can be downloaded at: https://www.mdpi.com/article/10.3390/foods13071031/s1, Figure S1: PS(+)MS full-scan of methanol extract from Sample 1 (beans), Figure S2: PS(+)MS full-scan of methanol extract from Sample 2 (toasted beans), Figure S3: PS(-)MS full-scan of methanol extract from Sample 1 (beans), Figure S4: PS(−)MS full-scan of methanol extract from Sample 2 (toasted beans), Figure S5: Product ion mass spectrum (MS/MS) of the ion of *m/z* 149 (ascribed as protonated serotonin), Figure S6: Product ion mass spectrum (MS/MS) of the ion of *m/z* 205 (ascribed as protonated I-Tryptophan), Figure S7: Product ion mass spectrum (MS/MS) of the ion of *m/z* 179 (ascribed as deprotonated Glucose), Figure S8: Product ion mass spectrum (MS/MS) of the ion of *m/z* 354 (ascribed as deprotonated caffeoyl tyrosine), Figure S9: Product ion mass spectrum (MS/MS) of the ion of *m/z* 433 (ascribed as deprotonated quercetin-3-O-arabinoside), Figure S10: Product ion mass spectrum (MS/MS) of the ion of *m/z* 461 (ascribed as deprotonated vanillic acid diglucoside), Figure S11: Product ion mass spectrum (MS/MS) of the ion of *m/z* 578 (ascribed as deprotonated apigenin-7-O-glucoside), Figure S12: Chromatogram generated for Sample 1 (beans), Figure S13: Chromatogram generated for Sample 2 (toasted beans), Figure S14: Mass spectrum generated for butane-2,3-diol, Figure S15: Mass spectrum generated for benzaldehyde, Figure S16: Mass spectrum generated for benzeneacetic, Figure S17: Mass spectrum generated for decanoic acid, Figure S18: Mass spectrum generated for dodecanoic acid, Figure S19: Mass spectrum generated for nonanoic acid, Figure S20: Mass spectrum generated for oleic acid, Figure S21: Mass spectrum generated for propanoic acid, Figure S22: Mass spectrum generated for pyrazine tetramethyl.
