# Supplementary material for: Influence of the Different Maturation Conditions of Cocoa Beans on the Chemical Profile of Craft Chocolates
Source: Foods. 2024 Mar 28;13(7):1031. doi: 10.3390/foods13071031 (PMC11011494; doi:10.3390/foods13071031)

Supplementary file

**Figure S1.** PS(+)MS full scan of methanol extract from Sample 1 (beans)

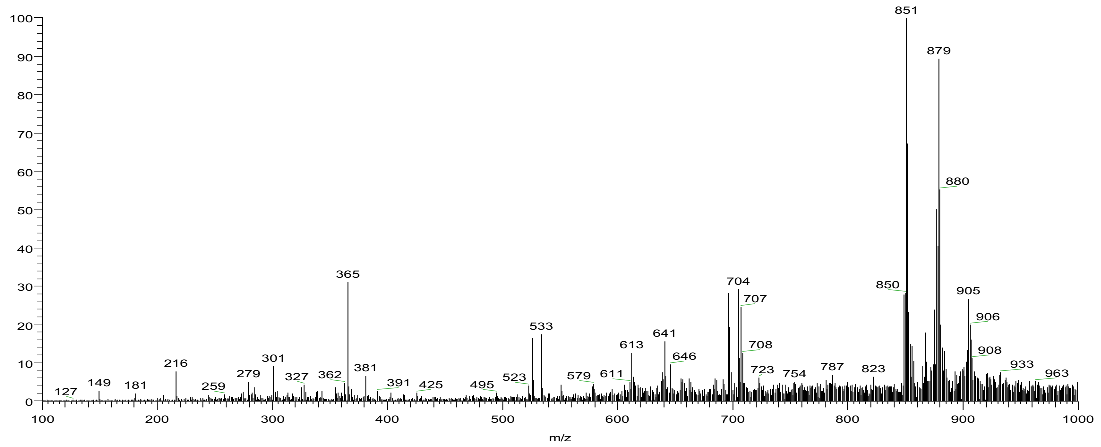


**Figure S2.** PS(+)MS full scan of methanol extract from Sample 2 (toasted beans)

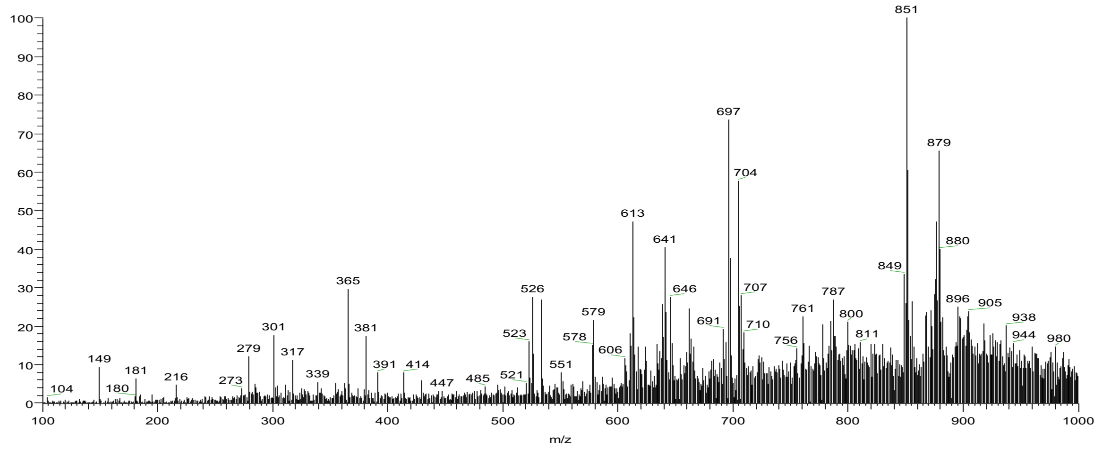


**Figure S3.** PS(-)MS full scan of methanol extract from Sample 1 (beans)

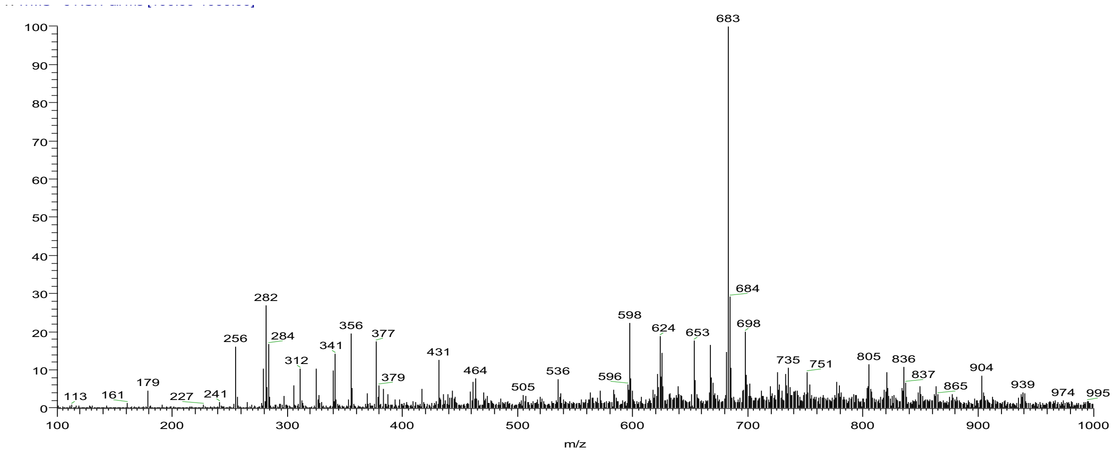

**Figure S4.** PS(-)MS full scan of methanol extract from Sample 2 (toasted beans)

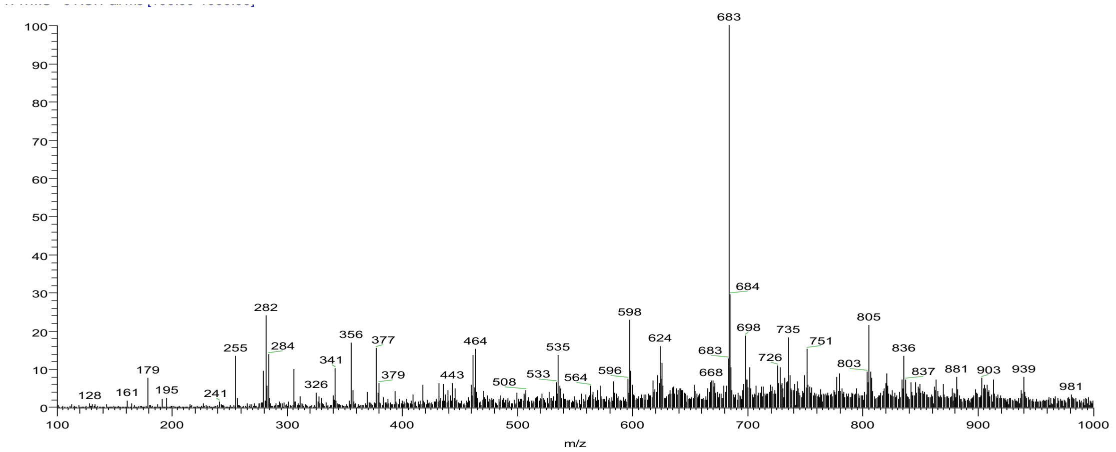


**Figure S5.** Product ion mass spectrum (MS/MS) of the ion of *m/z* 149 (ascribed as protonated Serotonin).

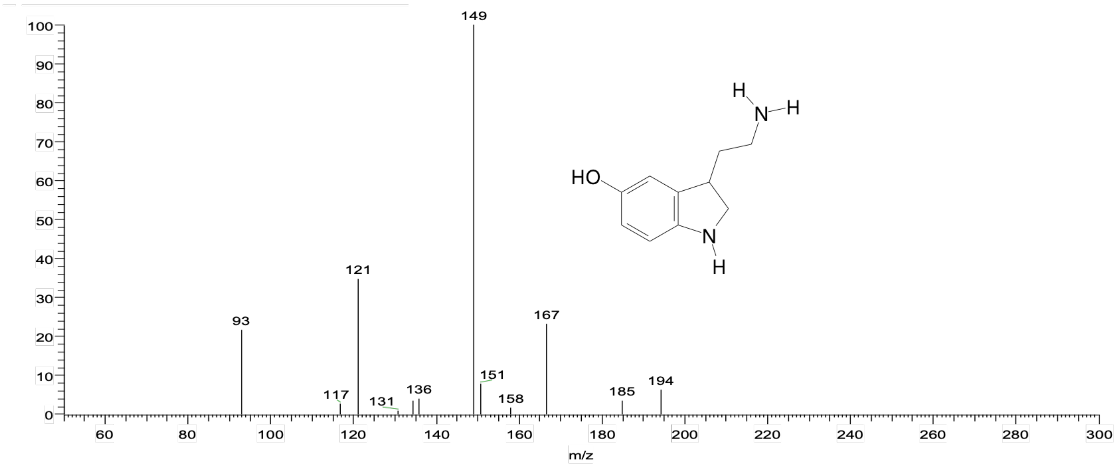


**Figure S6.** Product ion mass spectrum (MS/MS) of the ion of *m/z* 205 (ascribed as protonated l-Tryptophan).

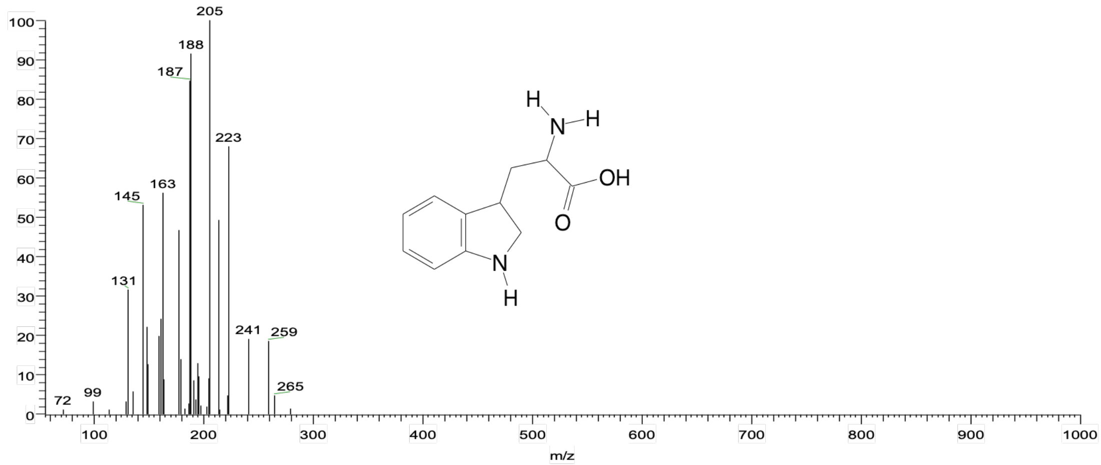

**Figure S7.** Product ion mass spectrum (MS/MS) of the ion of *m/z* 179 (ascribed as deprotonated Glucose).

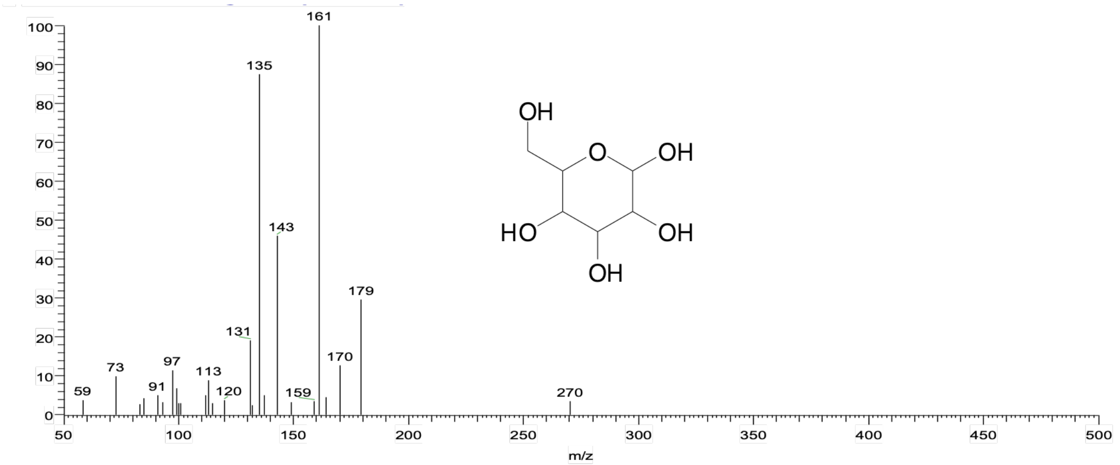


**Figure S8.** Product ion mass spectrum (MS/MS) of the ion of *m/z* 354 (ascribed as deprotonated Caffeoyl tyrosine).

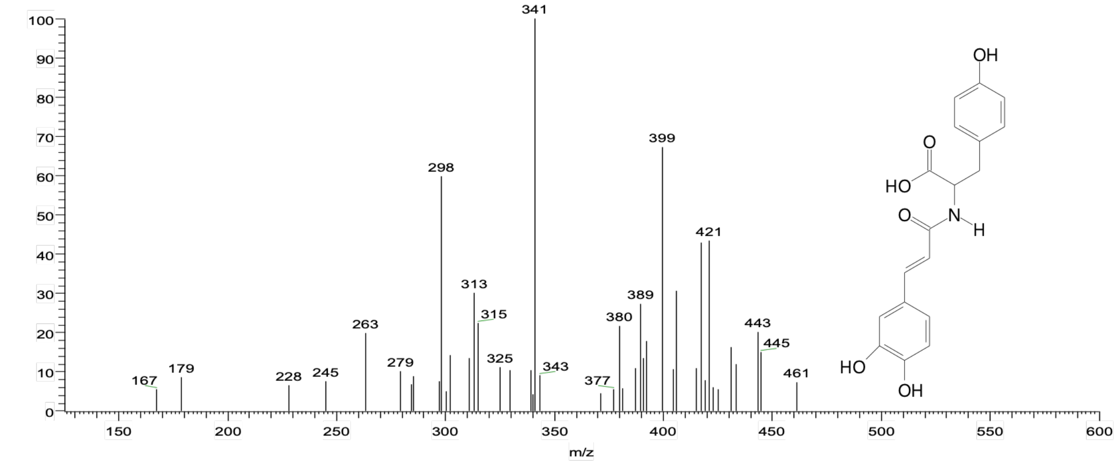


**Figure S9.** Product ion mass spectrum (MS/MS) of the ion of *m/z* 433 (ascribed as deprotonated Quercetin-3-*O*-arabinoside).

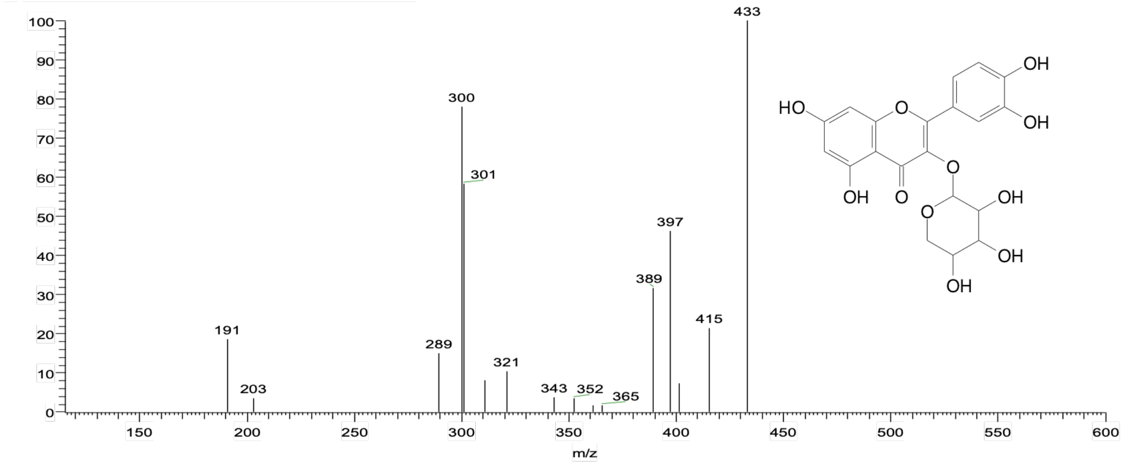

**Figure S10.** Product ion mass spectrum (MS/MS) of the ion of *m/z* 461 (ascribed as deprotonated Vanillic acid diglucoside).

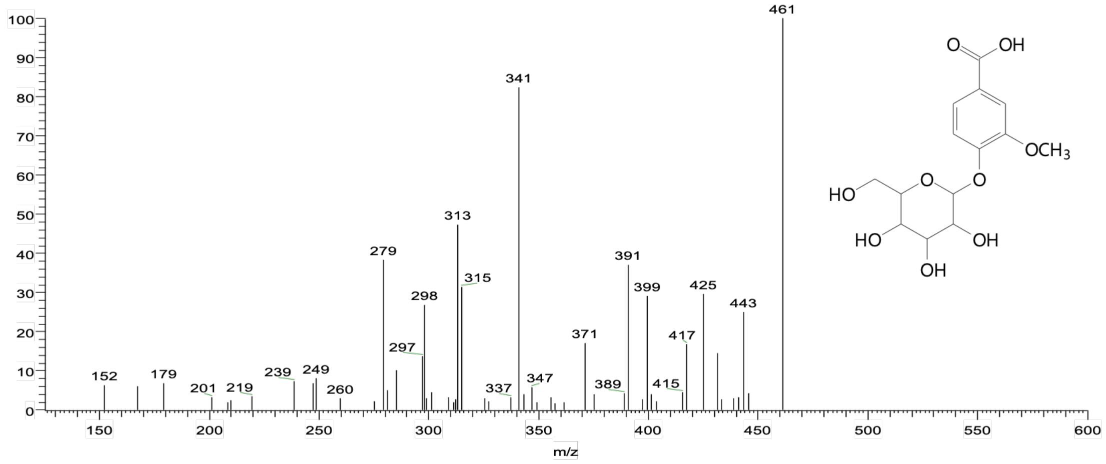


**Figure S11.** Product ion mass spectrum (MS/MS) of the ion of *m/z* 578 (ascribed as deprotonated Apigenin-7-*O*-glucoside).

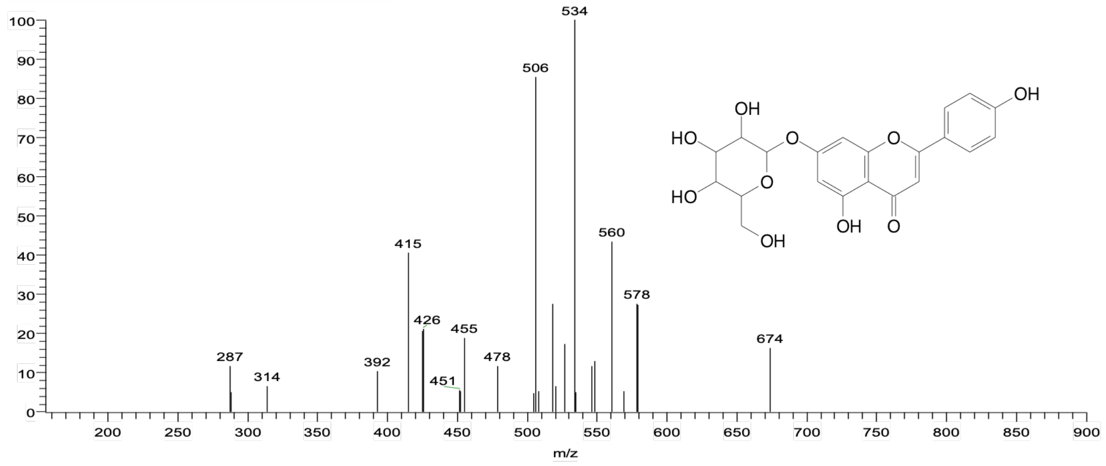


**Figure S12.** Chromatogram generated for Sample 1 (beans).

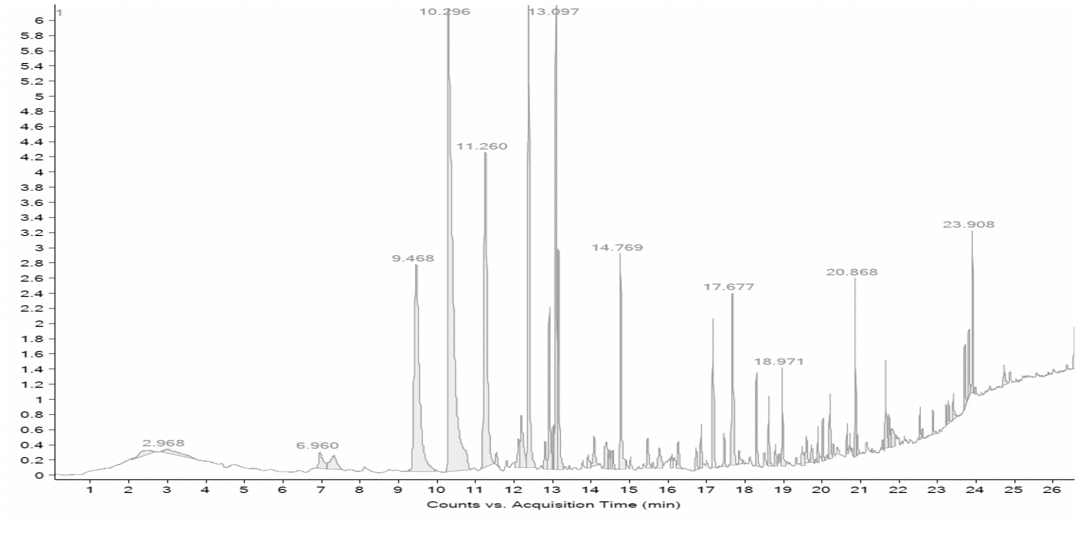

**Figure S13.** Chromatogram generated for Sample 2 (toasted beans).

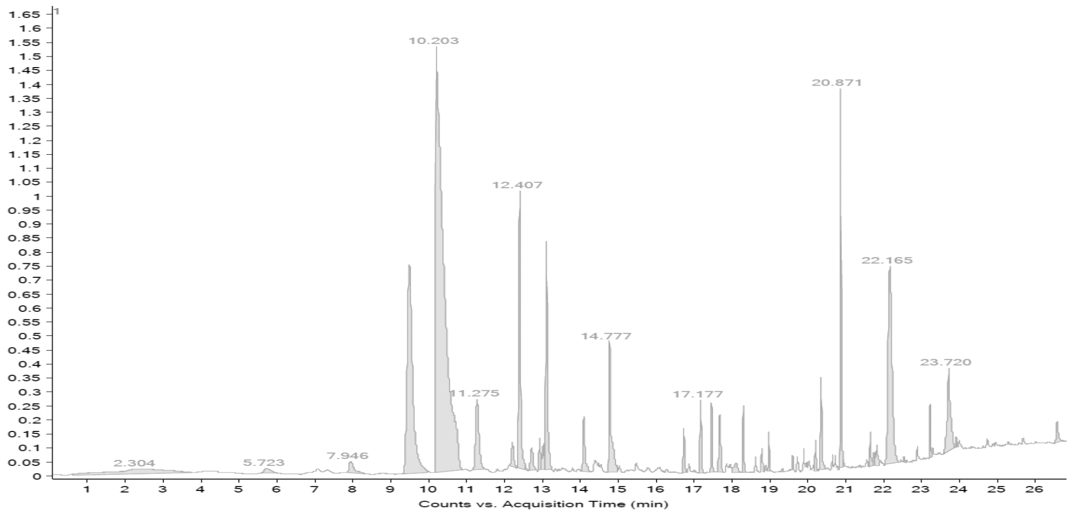


**Figure S14.** Mass spectrum generated for 2-3-Butanediol.

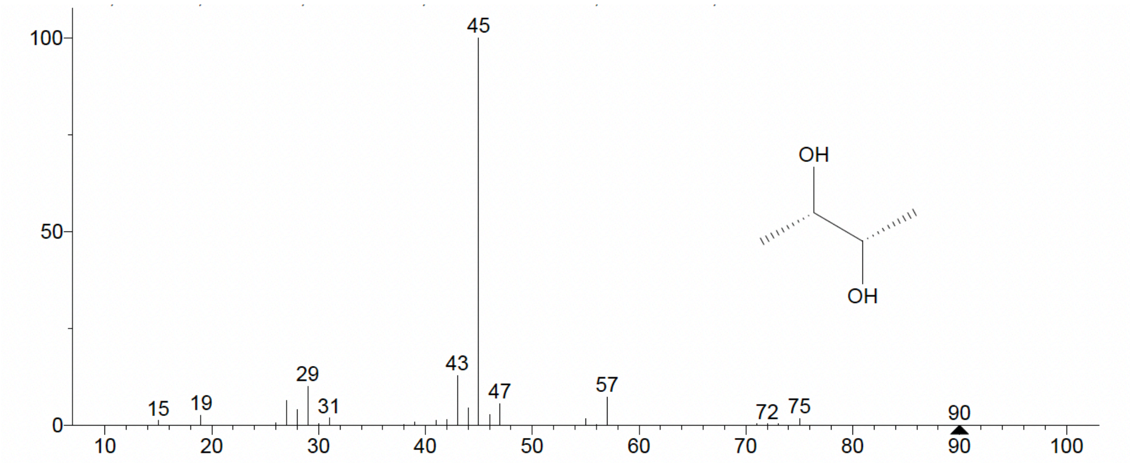


**Figure S15.** Mass spectrum generated for Benzaldehyde.

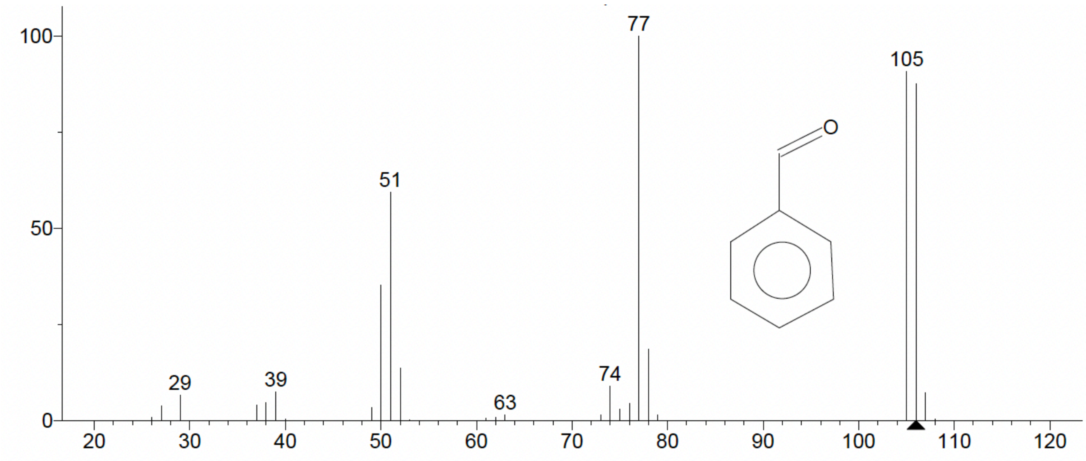

**Figure S16.** Mass spectrum generated for Benzeneacetic.

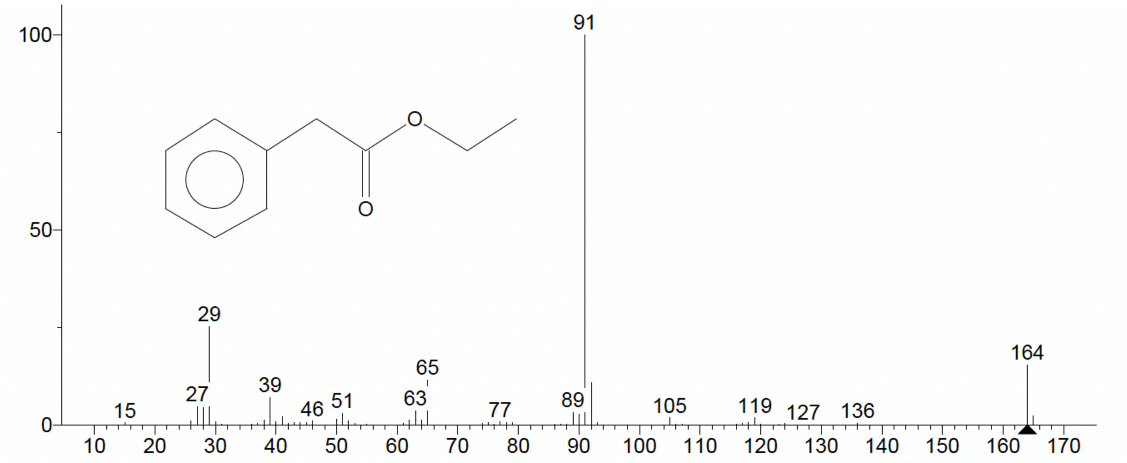


**Figure S17.** Mass spectrum generated for Decanoic acid.

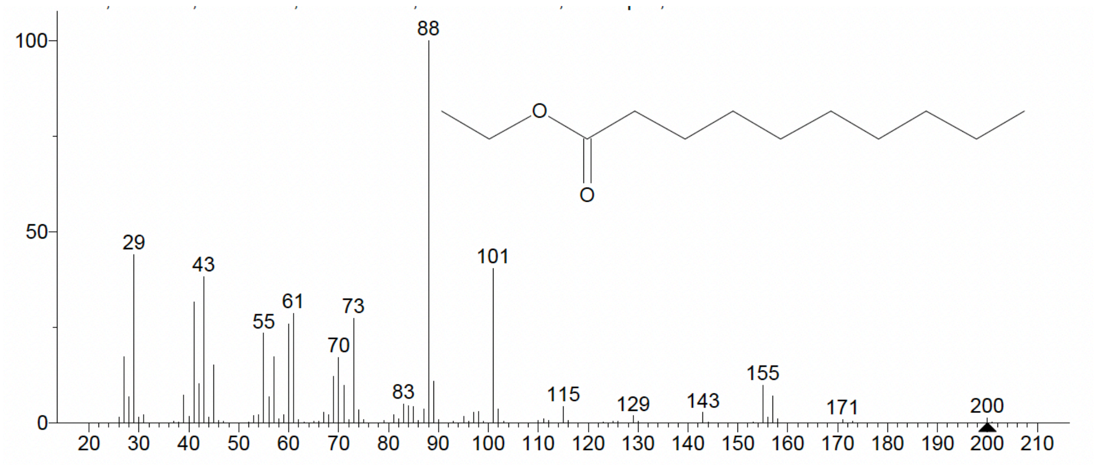


**Figure S18.** Mass spectrum generated for Dodecanoic acid.

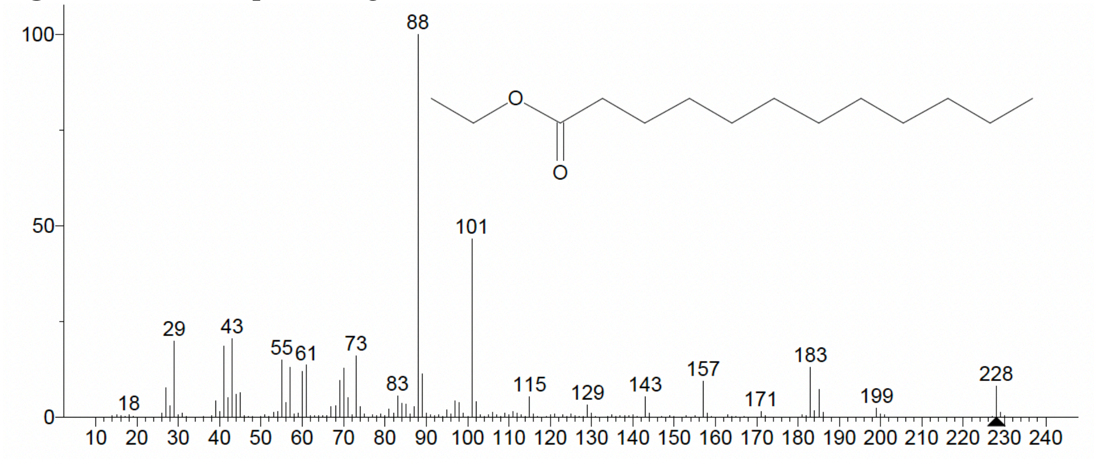

**Figure S19.** Mass spectrum generated for Nonanoic acid.

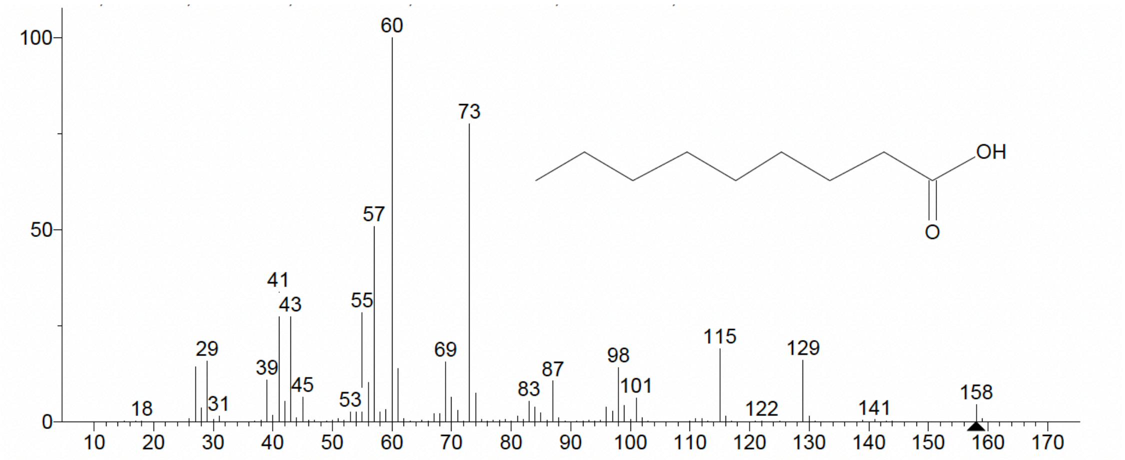


**Figure S20.** Mass spectrum generated for Oleic acid.

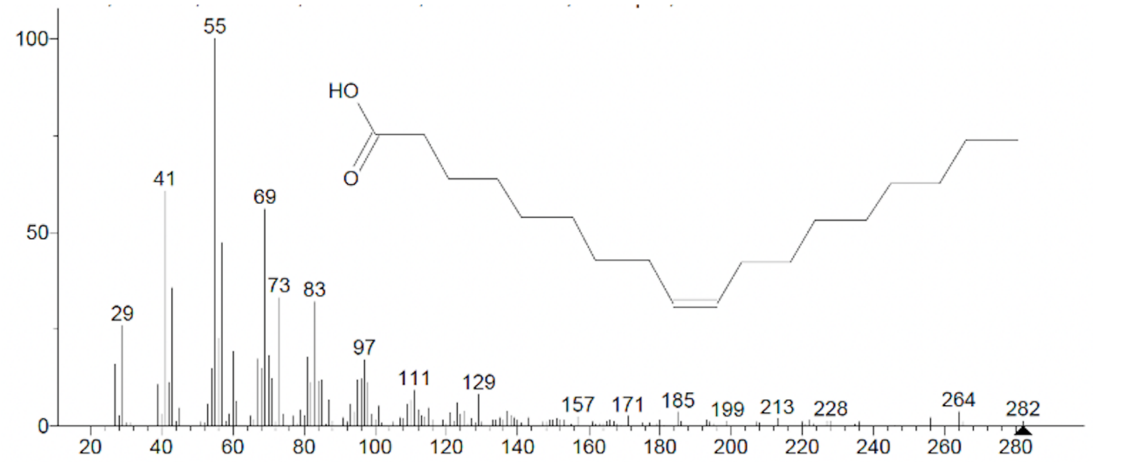


**Figure S21.** Mass spectrum generated for Propanoic acid.

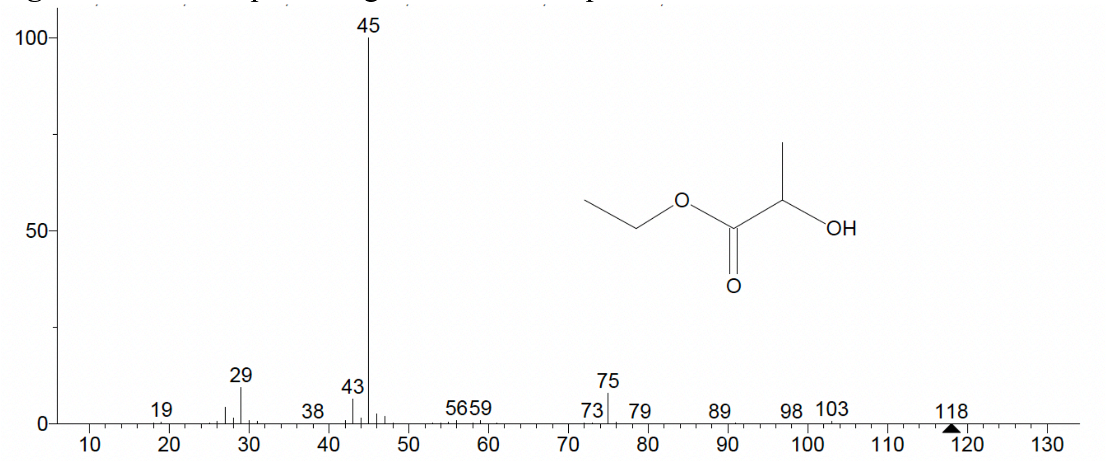

**Figure S22.** Mass spectrum generated for Pyrazine Tetramethyl.

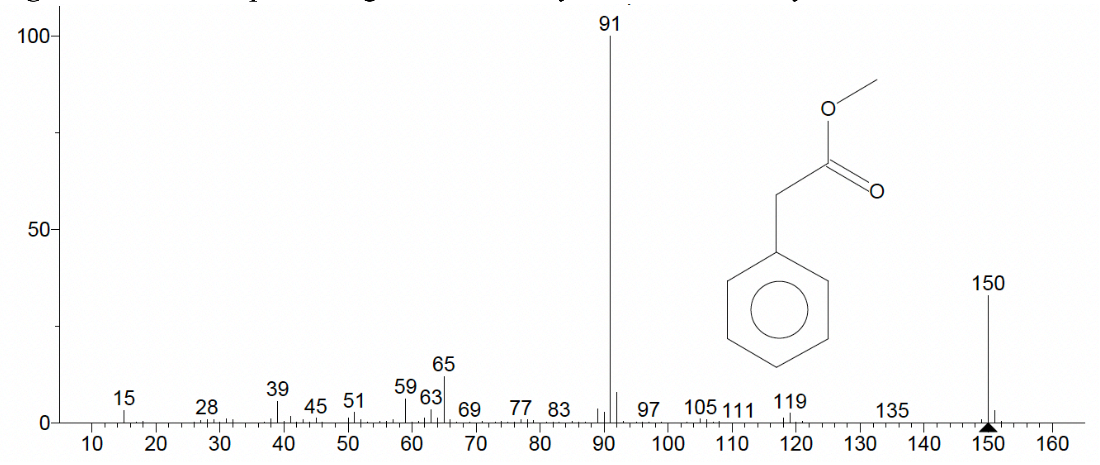

Supplement: Supplementary file 1 [file foods-13-01031-s001.zip › foods-2510352-supplementary.pdf]
